# Predictive correction of serum sodium concentration with formulas derived from the Edelman equation in patients with severe hyponatremia

**DOI:** 10.1038/s41598-023-28380-y

**Published:** 2023-01-31

**Authors:** Koya Nagase, Tsuyoshi Watanabe, Akihiro Nomura, Fumika N. Nagase, Keita Iwasaki, Yoshihiro Nakamura, Hiroki Ikai, Mari Yamamoto, Yukari Murai, Waka Yokoyama-Kokuryo, Naoho Takizawa, Hideaki Shimizu, Yoshiro Fujita

**Affiliations:** 1 Department of Nephrology, Chubu Rosai Hospital, 1-10-6, Komei-cho, Minato-ku, Nagoya, Aichi 455-8530 Japan; 2 Department of Rheumatology, Chubu Rosai Hospital, 1-10-6, Komei-cho, Minato-ku, Nagoya, Aichi 455-8530 Japan; 3 Department of Nephrology, Nagoya University Graduate School of Medicine, 65, Tsurumai-cho, Showa-ku, Nagoya, Aichi 466-8550 Japan; 4 Department of Nephrology and Renal Replacement, Daido Hospital, 9, Hakusui-cho, Minami-ku, Nagoya, Aichi 457-8511 Japan

**Keywords:** Nephrology, Kidney

## Abstract

Severe hyponatremia can cause life-threatening cerebral edema. Treatment comprises rapid elevation of serum sodium concentration; however, overcorrection can result in osmotic demyelination. This study investigated potential factors, including predictive correction based on the Edelman equation, associated with appropriate correction in 221 patients with a serum sodium concentration ≤ 120 mEq/L who were admitted to a hospital in Nagoya, Japan. Appropriate correction was defined as an elevation in serum sodium concentration in the range of 4–10 mEq/L in the first 24 h and within 18 mEq/L in the first 48 h after the start of the correction. Appropriate corrections were made in 132 (59.7%) of the 221 patients. Multivariate analysis revealed that predictive correction with an infusate and fluid loss formula derived from the Edelman equation was associated with appropriate correction of serum sodium concentration (adjusted odds ratio, 7.84; 95% confidence interval, 2.97–20.64). Relative without its use, the predictive equation results in a lower proportion of undercorrection (14.3% vs. 48.0%, respectively) and overcorrection (1.0% vs. 12.2%, respectively). These results suggest that predictive correction of serum sodium concentrations using the formula derived from the Edelman equation can play an essential role in the appropriate management of patients with severe hyponatremia.

## Introduction

Hyponatremia, defined as a serum sodium ([Na]) concentration below 135 mEq/L, is the most common electrolyte disorder, found in 30%–42% of hospitalized patients^1,2^, where the in-hospital mortality rate was reportedly 6.7%–7.1% among patients with severe hyponatremia (serum [Na] < 120 mEq/L)^3,4^. Although many cases are mild and relatively asymptomatic, profound hyponatremia can cause life-threatening cerebral edema, which requires prompt treatment to rapidly elevate serum [Na] levels^5,6^. Since there is some evidence that a correction of < 3–4 mEq/L within 24 h may be associated with excess mortality in patients with acute or postoperative hyponatremia^7,8^, expert opinion guidelines suggest a serum [Na] correction of ≥ 4 mEq/L within the first 24 h, even in patients with chronic hyponatremia^9^. However, overaggressive therapy can lead to a fatal neurological complication known as osmotic demyelination syndrome (ODS)^10–13^. Therefore, the American and European guidelines recommend that serum [Na] correction not exceed 10–12 mEq/L and 18 mEq/L within the first 24 and 48 h, respectively^9,14^. Due to this narrow therapeutic window, clinicians need to acquire a full picture regarding factors, including treatment strategies, which contribute to the appropriate correction of serum [Na] in patients with severe hyponatremia.

Recent guidelines and clinical trials have proposed treatments, such as hypertonic saline as a rapid intermittent bolus (RIB)^9,14^ and desmopressin use^15,16^, in patients with severe hyponatremia. However, due to the lack of evidence for the efficacy of these treatments, the safest and most effective strategy to treat severe hyponatremia remains unclear.

In an earlier study conducted among 98 patients with steady-state hyponatremia, Edelman et al. showed that plasma water [Na] is a function of exchangeable Na (Na_e_) and potassium (K_e_) divided by total body water (TBW)^17^.

1 $$Plasma \; \left[ {Na} \right] = 1.11\left( {Nae + Ke} \right)/TBW{-}25.6$$

Rose later suggested a simplified form of the Edelman equation for serum [Na], thereby making it more convenient to use in clinical practice^18^.

2 $$Serum \; [Na] = (Nae+Ke)/TBW$$

While these formulas may appear to be theoretically attractive for effective hyponatremia management, studies have suggested that they may not fully predict changes in serum [Na]^19,20^. However, the results of some previous studies have indicated that these formulas can make rough predictions of future changes in serum [Na]^21,22^.

This retrospective study aimed to investigate potential factors associated with the appropriate correction of severe hyponatremia. We focused on whether the predictive correction of serum [Na] with an infusate and fluid loss formula based on the Edelman equation, which accounted for a wide range of data sources pertaining to the input and output of Na, K, and water, would contribute to the establishment of the appropriate correction range recommended by existing guidelines.

## Results

### Patient characteristics and treatment outcomes

**Figure 1:**
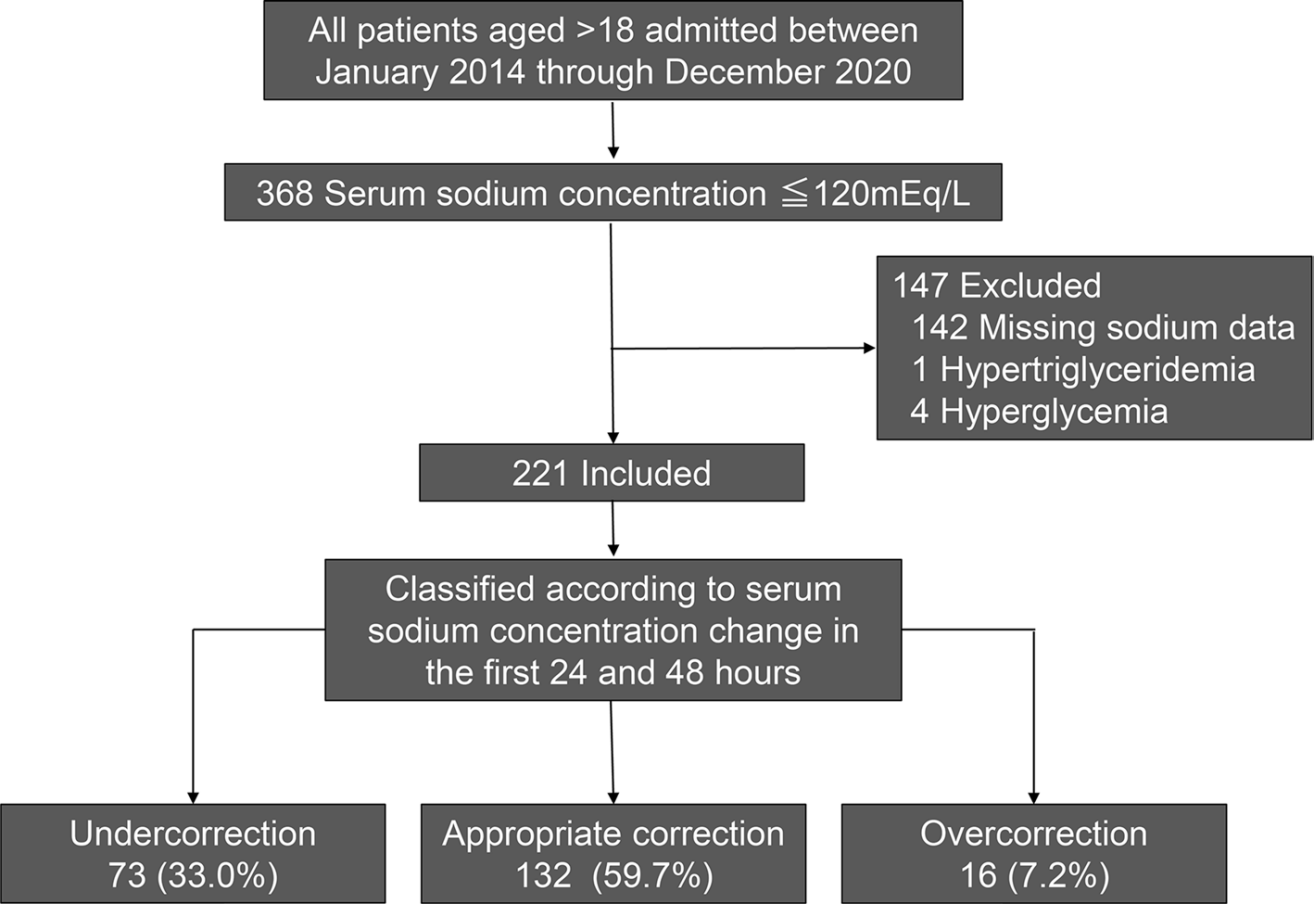
Patient selection process and subsequent categorization according to the degree of correction of hyponatremia.

A total of 368 patients were diagnosed with severe hyponatremia (serum [Na] level ≤ 120 mEq/L). Patients were excluded due to missing serum [Na] data within 12 h of the 24- and 48-h time points after the start of corrective treatment (n = 142), serum glucose concentrations of > 300 mg/dL (n = 4), and profound hypertriglyceridemia caused by acute pancreatitis (n = 1). Therefore, 221 patients were included in the main analysis (Fig. 1).

Table 1 shows the baseline characteristics of all patients. Appropriate serum [Na] corrections were achieved in 132 (59.7%) of 221 patients. The median changes in serum [Na] concentrations in the appropriate correction group were 6 mEq/L (interquartile range [IQR], 5–8) and 11 mEq/L (IQR, 8–13) at 24 and 48 h, respectively. The median changes in serum [Na] concentrations in the inappropriate correction group were 3 mEq/L (IQR, 1–3) and 6 mEq/L (IQR, 3–9) at 24 and 48 h, respectively. Univariate analyses indicated that patients in the appropriate correction group were more likely to have community-onset (61% vs. 42%); symptoms (81% vs. 60%); low Charlson Comorbidity Index (CCI) scores (1 vs. 2 points); low initial serum [Na] concentrations (117 vs. 118 mEq/L); high serum albumin concentrations (3.6 vs. 3.3 g/dL); used thiazide diuretics (20% vs. 6%); syndrome of inappropriate secretion of antidiuretic hormone (SIADH) (46% vs. 29%); and a drug-induced cause of hyponatremia (20% vs. 1%). Further, patients in the appropriate correction group were less likely to have solid tumor (20% vs. 31%), unidentified causes of hyponatremia (7% vs. 21%). Additionally, patients in the appropriate correction group were more likely to have been treated with predictive correction using the formula derived from the Edelman equation (62% vs. 17%), desmopressin (22% vs. 7%), or continuous hypertonic saline infusion (55% vs. 21%); they also underwent more serum [Na] assessments during the first 24 h (4 vs. 2 times) and 48 h (7 vs. 3 times), less likely to have been treated with isotonic saline (29% vs. 43%). Furthermore, the appropriate correction group had a lower mortality rate (8% vs. 19%) and a greater proportion of patients who received a consultation with a kidney specialist (81% vs. 52%).

**Table 1:** Baseline characteristics and outcomes of all patients, the appropriate correction, and inappropriate correction group with the serum sodium concentration ≤ 120 mEq/L.

	Total, n = 221	Appropriate correction group, n = 132	Inappropriate correction group, n = 89	*P* value
Age,yr median (IQR)	77 (68–83)	78 (67–84)	76 (69–81)	0.54
Women, n (%)	103 (47)	68 (51)	35 (39)	0.08
Community onset, n (%)	118 (53)	81 (61)	37 (42)	0.004
Body mass index, kg/m2 median (IQR), n = 214	20 (17–23)	20 (17–23)	19 (17–22)	0.11
Systolic BP, mm Hg median (IQR), n = 221	132 (114–150)	133 (115–149)	128 (111–152)	0.33
Diastolic BP, mm Hg median (IQR), n = 220	72 (62–86)	72 (64–86)	74 (60–85)	0.77
Symptomatic, n (%)	160 (72)	107 (81)	53 (60)	< .001
Severe symptom, n (%)	46 (21)	33 (25)	13 (15)	0.06
Moderate symptom, n (%)	114 (52)	74 (56)	40 (45)	0.11
Comorbidities, n (%)				
Myocardial infarction	7 (3.2)	4 (3)	3 (3)	1
Congestive heart failure	31 (14)	17 (13)	14 (16)	0.55
Cerebrovascular disease	16 (7)	9 (7)	7 (8)	0.77
Dementia	17 (8)	12 (9)	5 (6)	0.34
Connective tissue disease	11 (5)	6 (5)	5 (6)	0.76
Diabetes	53 (24)	31 (23)	22 (25)	0.83
Chronic kidney disease	36 (16)	19 (14)	17 (19)	0.35
Solid tumor	54 (24)	26 (20)	28 (31)	0.046
Charlson comorbidity index				
Score, median (IQR)	1 (0–3)	1 (0–3)	2 (1–4)	0.01
0, n (%)	64 (29)	45 (34)	19 (21)	0.04
1, n (%)	51 (23)	32 (24)	19 (21)	0.62
2, n (%)	41 (19)	21 (16)	20 (22)	0.22
≧3, n (%)	65 (29)	34 (26)	31 (35)	0.15
Laboratory values at bottom sodium level, median (IQR)				
Sodium, mEq/L,n = 221	117 (114–119)	117 (113–118)	118 (115–119)	0.02
Potassium, mEq/L, n = 221	4.2 (3.8–4.8)	4.3 (3.9–4.9)	4.2 (3.7–4.8)	0.6
Phosphorus, mg/dL, n = 101	2.9 (2.4–3.4)	2.9 (2.4–3.6)	2.9 (2.4–3.3)	0.34
Magnesium, mg/dL, n = 67	1.9 (1.7–2.1)	1.9 (1.7–2.1)	1.8 (1.7–2.1)	0.1
Creatinine, mg/dL, n = 220	0.64 (0.46–1.02)	0.63 (0.47–0.99)	0.64 (0.45–1.09)	0.72
eGFR, ml/min/1.73m2 , n = 220	81 (48–112)	81 (54–111)	81 (38–113)	0.84
Uric acid, mg/dL, n = 164	2.9 (2.2–5.1)	2.9 (2.4–5.0)	2.9 (1.9–5.3)	0.69
Serum osmolarity, mOsm/kg, n = 173	242 (235–248)	241 (234–248)	244 (239–248)	0.08
Albumin, g/dL, n = 198	3.5 (2.9–4.0)	3.6 (3.1–4.0)	3.3 (2.6–3.8)	0.003
Glucose, mg/dL, n = 191	122 (107–151)	121 (109–150)	123 (102–151)	0.78
Urine sodium, mEq/L, n = 187	62 (28–94)	63 (28–94)	58 (28–92)	0.56
Urine potassium, mEq/L, n = 187	28 (18–41)	30 (20–40)	26 (15–44)	0.24
Urine osmolality, mEq/L, n = 171	408 (291–500)	407 (290–486)	427 (314–516)	0.46
Cause of hyponatremia, n (%)				
Primary polydipsia	15 (7)	10 (8)	5 (6)	0.57
Hypovolemic	84 (38)	50 (38)	34 (38)	0.96
SIADH	87 (39)	61 (46)	26 (29)	0.01
Adrenal insufficiency	3 (1)	2 (2)	1 (1)	1
Drug induced	27 (12)	26 (20)	1 (1)	< .001
Heart failure	14 (6)	7 (5)	7 (8)	0.44
Unidentified cause	28 (13)	9 (7)	19 (21)	0.01
Daily use medication, n (%)				
Thiazide diuretics	32 (15)	27 (20)	5 (6)	0.002
Loop diuretics	36 (16)	20 (15)	16 (18)	0.58
Aldosterone antagonists	14 (6)	10 (8)	4 (4)	0.36
NSAIDs	33 (15)	19 (14)	14 (16)	0.79
SSRI	9 (4)	7 (5)	2 (2)	0.32
Antiseizure medication	9 (4)	6 (5)	3 (3)	0.74
Antipsychotic medication	10 (5)	9 (7)	1 (1)	0.052
Correction method				
The formula derived from the Edelman equation, n (%)	98 (44)	83 (62)	15 (17)	< .001
Number of measurements of serum sodium level, median during the first 24 h (IQR)	4 (2–5)	4 (3–6)	2 (2–4)	< .001
Number of measurements of serum sodium level, median during the first 48 h (IQR)	6 (3–9)	7 (5–9)	3 (3–6)	< .001
Isotonic saline, n (%)	76 (34)	38 (29)	38 (43)	0.03
Hypertonic saline bolus infusion, n (%)	14 (6)	11 (8)	3 (3)	0.14
Hypertonic saline continuous infusion, n (%)	92 (42)	73 (55)	19 (21)	< .001
Electrolyte repletion, n (%)	26 (12)	14 (11)	12 (13)	0.52
Loop diuretics, n (%)	9 (4)	5 (4)	4 (4)	1
Vaptans, n (%)	6 (3)	3 (2)	3 (3)	0.69
Desmopressin, n (%)	35 (16)	29 (22)	6 (7)	0.002
Kidney specialist intervention, n (%)	153 (69)	107 (81)	46 (52)	< .001
Outcome, n (%)				
Length of hospital stay (IQR)	28 (14–52)	25 (12–51)	30 (18–52)	0.29
ODS	0(0)	0 (0)	0 (0)	
Mortality	27 (12)	10 (8)	17 (19)	0.01

*IQR* interquartile range; *BP* blood pressure; *eGFR* estimated glomerular filtration rate; *SIADH* syndrome of inappropriate secretion of antidiuretic hormone; *NSAIDs* non-steroidal anti-inflammatory drugs; *SSRI* selective serotonin reuptake inhibitors; *ODS* osmotic demyelination syndrome

Categorical variables are shown as numbers (percentages) and continuous variables as medians (25–75 percentiles)

*P* values represent a significant difference between appropriate and inappropriate correction group.

The multivariate analysis indicated that higher BMI (adjusted odds ratio, 1.16; 95% confidence interval, 1.03–1.32), higher serum [K] (adjusted odds ratio, 1.84; 95% confidence interval, 1.08–3.14), the application of predictive correction with an infusate and fluid loss formula based on the Edelman equation (adjusted odds ratio, 7.84; 95% confidence interval, 2.97–20.64), and hypertonic saline continuous infusion (adjusted odds ratio, 4.15; 95% confidence interval, 1.32–13.03) were variables associated with appropriate serum [Na] correction (Table 2). Since the inappropriate correction group includes two extreme groups (undercorrection and overcorrection), we performed a separate analysis of undercorrection versus non-undercorrection and overcorrection versus non-overcorrection for further sensitivity analysis (Supplementary Tables 1, 2, 3, 4). The multivariate analysis indicated that the predictive formula use (adjusted odds ratio, 4.13; 95% confidence interval, 1.53–11.13) was associated with non-undercorrection (Supplementary Table 2), and the predictive formula use (adjusted odds ratio, 74.62; 95% confidence interval, 2.29–2433.81) and high initial serum [Na] (adjusted odds ratio, 1.54; 95% confidence interval, 1.07–2.22) were associated with non-overcorrection (Supplementary Table 4).

**Table 2:** Factors associated with appropriate correction of serum sodium concentration in patients with the serum sodium concentration ≤ 120 mEq/L.

Variables	Adjusted OR	95% CI	*p* value
Age	0.97	0.93–1.02	0.21
Women	1.96	0.74–5.20	0.18
BMI	1.16	1.03–1.32	0.01
Solid tumor	1.71	0.31–9.42	0.53
Charlson Comorbidity Index Score	0.77	0.56–1.08	0.13
Laboratory values at bottom sodium level			
Serum sodium	1	0.87–1.13	0.9
Serum pottasium	1.84	1.08–3.14	0.03
Urine sodium	1	0.99–1.00	0.17
Urine osmolarity	1	1.00–1.00	0.54
Cause of hyponatremia			
Drug induced	8.83	0.60–130.28	0.11
Unidentified cause	0.8	0.17–3.84	0.78
Daily use medication			
Thiazide diuretics	1.01	0.12–8.19	0.99
Correction method			
The formula derived from the Edelman equation	7.84	2.97–20.64	< .001
Number of measurements of serum sodium level during the first 24 h	1.46	0.88–2.4	0.14
Number of measurements of serum sodium level during the first 48 h	0.86	0.66–1.12	0.25
Isotonic saline	1.6	0.50–5.08	0.43
Hypertonic saline bolus infusion	0.9	0.13–5.99	0.91
Hypertonic saline continuous infusion	4.15	1.32–13.03	0.02
Electrolyte repletion	0.62	0.14–2.71	0.53
Desmopressin	1.03	0.21–5.01	0.97
Kidney specialist intervention	0.73	0.21–2.49	0.61

*OR* odds ratio; 95% CI, 95% confidence interval.

### Analysis of the patients with severe hyponatremia treated using the Edelman predictive equation

**Figure 2:**
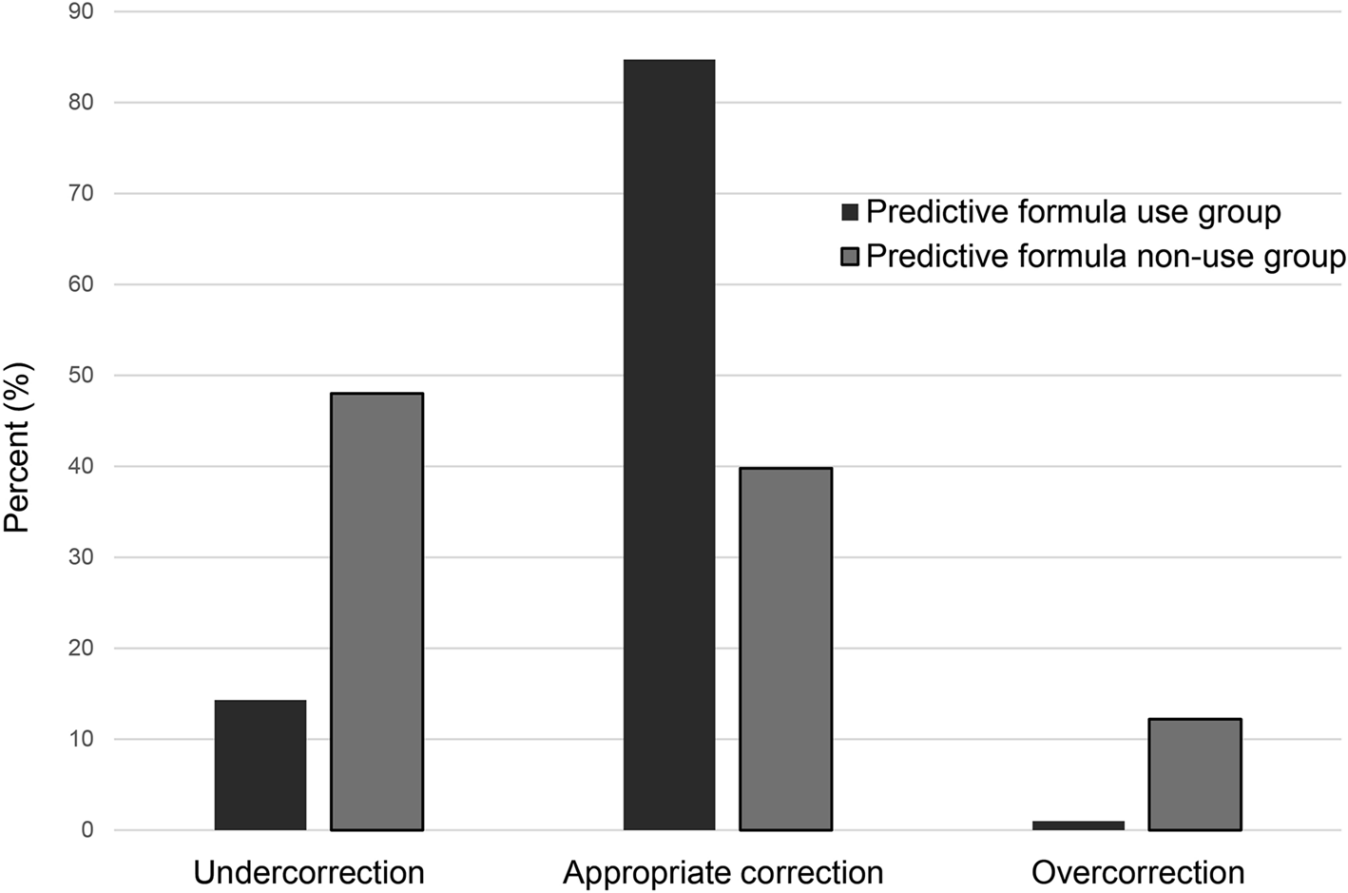
Percentage of patients with undercorrection, appropriate correction, or overcorrection with an initial serum sodium concentration ≤ 120 mEq/L according to the use or non-use of the prediction formula derived from the Edelman equation.

**Figure 3:**
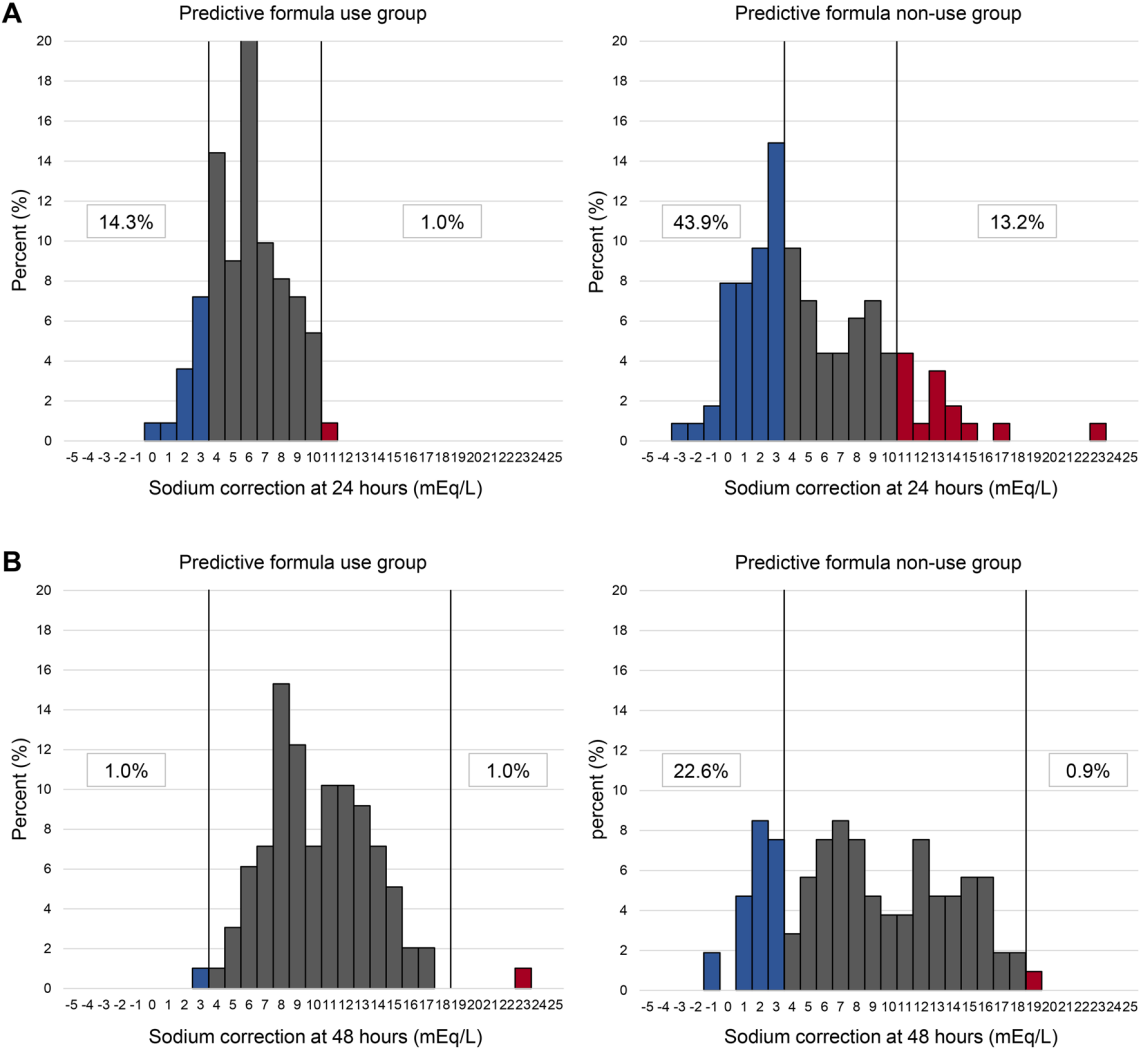
Distribution of the change in the initial serum sodium concentration ≤ 120 mEq/L at baseline to 24 and 48 h (**A** and **B**) in patients for whom the predictive equation was used and not used, and the percentage of patients with inappropriate correction.

The predictive correction with the formula derived from the Edelman equation was applied to correct hyponatremia in a total of 98 (44.3%) patients in this study. The percentages of patients with undercorrection, appropriate correction, and overcorrection were 14.3%, 84.7%, and 1.0% in the predictive equation use group, respectively (Fig. 2); while, those with undercorrection, appropriate correction, and overcorrection were 48.0%, 39.8%, and 12.2% in the predictive equation non-use group, respectively. The detailed percentages of patients with undercorrection, appropriate correction, and overcorrection at 24 and 48 h after treatment for hyponatremia are shown in Fig. 3.

## Discussion

This retrospective study evaluated novel factors potentially associated with the appropriate correction of profound hyponatremia. Multivariate analysis demonstrated the efficacy of an infusate and fluid loss formula derived from the Edelman equation for hyponatremia correction. The use of the predictive equation resulted in an appropriate correction in a greater proportion of patients (84.7%), as well as lower rates of both undercorrection (14.3%) and overcorrection (1.0%). High BMI and high serum [K] concentrations, for which low BMI and low serum [K] were known as risk factors for overcorrection, were also significantly associated with the appropriate correction of hyponatremia in this study. However, younger age, female sex, solid tumor, low CCI, low urine [Na], and low urine osmolality were not significantly associated with the appropriate correction of hyponatremia^23–25^. In addition, the administration of hypertonic saline continuous infusion was also associated with the appropriate correction of hyponatremia.

In our correction with the infusate and fluid loss formula derived from the Edelman equation, future serum [Na] was predicted using Eq. (3):

3 $$Serum [Na]2 = (serum [Na]1\times TBW1+\Delta [Na+K])/(TBW1+\Delta TBW)$$

We used the simplified formula by Rose BD for ease of use by physicians at the bedside. Although it lacks adequate scientific rigor, the simplified formula by Rose BD can estimate the change of serum [Na] using the data for electrolyte and fluid balance with the same precision as the Edelman Equation^21,22^.

While expert opinions regarding the theoretical performance and limitations of predictive formulas for the appropriate treatment of profound hyponatremia have been proposed^20,21^, few studies have sought to provide clinical evidence to substantiate these claims. The most distinctive feature of this study is that our use of the predictive equation accounted for a wide range of data sources pertaining to the input and output of Na, K, and water. This allowed us to optimize the accuracy of serum [Na] prediction. Moreover, the amount of urine flow per hour and serum [Na], serum [K], urine [Na], and urine [K] concentrations were measured on an average of 5.1 and 8.6 times at 24 and 48 h, respectively, in patients for whom the predictive equation was used. Furthermore, we frequently calculated the future serum [Na] to fall within the intended appropriate correction range. Previous studies have demonstrated the clinical utility and efficacy of the infusate formula for future serum [Na] prediction in patients with hyponatremia despite the absence of information on the concomitant loss of urine and other fluids^5,26^. Nevertheless, to obtain a more theoretically accurate correction, we applied the infusate and fluid loss formula to account for a range of variables, such as fluid therapy type, substantial aquaresis, and extrarenal fluid and Na losses^5^.

Although recent American and European guidelines have recommended the administration of RIB hypertonic saline to achieve an early increase in serum [Na]^9,14^, only 6.3% of patients with hyponatremia were treated with RIB hypertonic saline infusion in this study. This is likely because, until recently, there has been a lack of clinical studies that have substantiated the effects of RIB therapy with hypertonic saline. Recent studies, including randomized controlled trials, have indicated that RIB therapy with hypertonic saline can rapidly and safely correct hyponatremia^27,28^. However, overcorrection occurred in 17.2%–19.4% of patients administered RIB and 24.2%–26.0% of patients who received a slow continuous infusion of hypertonic saline^27^.

Although direct comparisons to the present study are difficult, our results suggest that the predictive correction with an infusate and fluid loss formula derived from the Edelman equation may reduce overcorrection compared with RIB therapy. The advantage of predictive correction is that it monitors urine volume and facilitates a quick response to the development of sudden substantial water diuresis, which is the most common reason for hyponatremia overcorrection^29,30^. The development of sudden substantial water diuresis can be counteracted by administering water or desmopressin; an infusate and fluid loss formula based on the Edelman equation can then be used to predict future serum [Na] concentrations.

Undercorrection was observed in 14.3% of patients for whom the predictive equation was used. Although the majority of previous studies have rarely evaluated undercorrection as an outcome, existing evidence suggests an association between undercorrection and increased mortality; therefore, undercorrection should be considered a correction failure^7,8,31^. Although we subsequently performed the sensitivity and multivariate analysis to investigate the potential factor associated with undercorrection exclusively within the Edelman equation use group, we could not identify the significant variable (data not shown). Although we also considered that the lower rate of application of RIB hypertonic saline could contribute to undercorrection of hyponatremia, it did not become a potential factor for undercorrection of hyponatremia in that multivariate analysis. A further study is required to identify the potential factors associated with correction failure of hyponatremia using predictive correction with the formula derived from the Edelman equation.

The advantages of RIB therapy with hypertonic saline include secure and rapid partial serum [Na] correction, which prevents complications due to cerebral edema^32^. Therefore, RIB therapy with hypertonic saline may be more effective as an initial treatment for hyponatremia than predictive serum [Na] correction using the formula. Current guidelines do not provide recommendations on how to administer continuous fluid infusion for the fine-tuning of serum [Na] following initial RIB therapy. We propose that the application of predictive correction with the Edelman equation following RIB therapy should theoretically prevent the overcorrection of serum [Na].

Previous clinical and physiological studies have indicated that the infusate and fluid loss formula, which accounts for urine volume and other fluid and salt losses, cannot predict changes in serum [Na] concentration with complete accuracy^19–21^. This may be attributed to two factors. First, regulatory volume decreases with the cellular outflow of K, chloride, and organic osmolytes under hypotonic stress; this reduces serum [Na] without changing TBW or the amount of exchangeable Na and K^22,33,34^. Second, the Edelman equation is based on steady-state observations and may not account for the exchange between osmotic [Na] (serum [Na]) and non-osmotic Na (Na bound to glycosaminoglycans in the bone and cartilage)^19,22^. The physiological regulation of osmotically inactive Na and its response to changes in osmotically active [Na] remain largely unknown, and it is unclear to what extent serum [Na] concentrations are affected in clinically problematic ranges^35^. Although we showed that predictive correction of severe hyponatremia with the Edelman equation is valuable as a guide for appropriate correction in this study, we also should keep in mind these theoretical and pathophysiological limitations.

Several limitations are acknowledged in our study. First, as this study used a retrospective design, the observed associations between the appropriate correction of hyponatremia and the predictor variables could not be interpreted as causal relationships. Second, although we included kidney specialist intervention and number of serum [Na] measurements as covariates in the multivariate analysis, users of the correction equation may be better prepared to treat patients with hyponatremia. It is possible that enthusiasm and proficiency for the treatment of hyponatremia influence treatment outcomes. Third, this was a single-center study. Therefore, caution should be exercised when generalizing the results to other geographic regions or patients in the community. Fourth, although we made comprehensive estimations of water and electrolyte input and output when using the formula based on the Edelman equation, it is possible that these values may not have been completely accurate. Fifth, several patients for whom the predictive equation was not used lacked urinary electrolyte and osmolality data; therefore, this may have affected the accuracy of the determination of hyponatremia etiology. Finally, there is currently a lack of strong evidence regarding the appropriate correction value, especially the lower limit; this may change with future findings.

In conclusion, this retrospective study suggests that the predictive correction of serum [Na] concentrations using an infusate and fluid loss formula derived from the Edelman equation can play an integral role in the appropriate management of patients with severe hyponatremia. Prospective controlled studies are required to establish causality between predictive correction and the appropriate correction of hyponatremia.

## Methods

### Inclusion criteria, definition of appropriate correction, and outcome

This retrospective study was conducted at a teaching hospital in Nagoya, Japan, between January 2014 and December 2020. The study protocol was designed following the Helsinki declaration and approved by the Clinical Research Ethics Committee of Chubu Rosai Hospital (reference no. 202103–04, approved on April 20, 2021). As the study data were anonymized before use and did not lead to patient identification, the requirement for informed consent was waived by the Clinical Research Ethics Committee of Chubu Rosai Hospital.

We included adults aged > 18 years who had a serum [Na] level ≤ 120 mEq/L, as these patients are at risk of ODS^9^. We excluded patients who had no available data pertaining to serum [Na] within 12 h of the 24- and/or 48-h time points after initiating the corrective treatment because the appropriateness of serum [Na] correction could not be determined among those patients. Furthermore, we excluded patients with serum glucose levels of > 300 mg/dL on admission and those with hypertriglyceridemia presenting with pseudohyponatremia. For each study participant, we estimated serum [Na] level at 24 h using the following formula: s[Na] = Naa + [(Nab—Naa) × (24—Ta)/Tb × Ta)], where Naa and Ta are the nearest serum sodium and time values before 24 h mark, respectively, and Nab and Tb are the nearest serum sodium and time value after 24 h mark, respectively ^31^. We estimated serum [Na] level at 48 h in the same manner as at 24 h. Following previous guidelines, appropriate correction was defined as a change in [Na] in the range of 4–10 mEg/L in the first 24 h and within 18 mEq/L in the first 48 h after corrective treatment initiation, respectively^9,14^. Overcorrection was defined as an increase in the serum [Na] level > 10 or 18 mmol/L within 24 or 48 h; undercorrection was defined as a [Na] correction of < 4 mEq/L in the first 24 h in this study.

Our primary analysis was to investigate potential factors associated with the appropriate correction of severe hyponatremia. The main outcome was the achievement of appropriate serum [Na] correction.

### Baseline data collection

Patient data were collected by reviewing electronic medical records. Demographic data included age, sex, community-onset or not at the development of severe hyponatremia, comorbidities (myocardial infarction, congestive heart failure, cerebrovascular disease, dementia, connective tissue disease, diabetes, chronic kidney disease, solid tumor), daily medication use (thiazide and loop diuretics, aldosterone antagonists, nonsteroidal anti-inflammatory drugs, selective serotonin reuptake inhibitors, antiseizure drugs, and antipsychotic drugs), and cause of hyponatremia (primary polydipsia, hypovolemia, SIADH, adrenal insufficiency, drug-induced hyponatremia, heart failure, and unknown cause). We diagnosed SIAD based on the following criteria (an inciting factor, plasma osmolality < 275 mOsm/kg, urine [Na] > 30 mmol/L, urine osmolality > 100 mOsm/kg, clinical euvolemia, absence of diuretic use, and normal thyrotropin and adrenal glucocorticoid secretion)^9^. Moreover, we diagnosed primary polydipsia, hypovolemia, adrenal insufficiency, drug-induced hyponatremia, and heart failure based on symptoms, clinical volume status, past medical history, laboratory data, and imaging findings. Comorbidity severity was recorded according to the CCI^36^. Anthropometric and clinical data included BMI, systolic and diastolic blood pressure, and the following laboratory parameters: serum [Na], serum [K], phosphorus, magnesium, creatinine, albumin, glucose, urine [Na], urine [K], estimated glomerular filtration rate, and serum and urine osmolality. Symptomatic or asymptomatic status was also recorded. Symptoms of hyponatremia were further categorized as moderate (nausea without vomiting, headache, drowsiness, confusion, general weakness, and malaise) or severe (vomiting, seizure, and coma [Glasgow Coma Scale score ≤ 8]) following previous studies and guidelines^14,27^.

### Correction method

We reviewed the correction method (predictive correction for future serum [Na] using the formula based on the Edelman equation, isotonic saline, hypertonic saline [bolus or continuous infusion], loop diuretics, vasopressin receptor antagonists, desmopressin, and intravenous or oral electrolyte repletion [including potassium (K), magnesium, calcium, and phosphorus]), and the number of serum [Na] measurements within 24 and 48 h after corrective therapy initiation. Cases that were administered the target infusate volume calculated using the predictive formula, as documented by the physicians, were allocated to the predictive equation use group. Regarding the predictive correction for future serum [Na], we used Eq. (2)^18^. To determine the appropriate fluid composition and volume that corresponded to substantial urine loss, we applied the following infusate and fluid loss formula based on the Edelman Equation^22,37^:

3 $$Serum {[Na]}_{2} = (serum {[Na]}_{1}\times TBW1+\Delta [Na+K])/(TBW1+\Delta TBW)$$

Here, serum [Na]_1_ represents the current serum [Na], and serum [Na]_2_ represents the future serum [Na]. All data sources pertaining to the input and output of Na, K, and water (including infusion, urine, food, drink, drainage [Na, K, and water], electrolyte repletion [Na, K], and insensible evaporation [water]) were collected to optimize the accuracy of serum [Na]2 predictions via Eq. (3) (Supplementary Fig. 1).

After the diagnosis of hyponatremia, the output of Na, K, and water was calculated by examining spot urine and estimating the loss volume of urine and insensible excretion. We then calculated how much serum [Na] would change by taking Na, K, and water orally or intravenously and initiated treatment to achieve the target correction rate. To predict the future urine output of Na, K, and water, we used data from the most recent time point for urine [Na], urine [K], and the amount of urine flow, as we also concurrently performed spot urine tests for each blood test. In the case of diuretic therapy for hypervolemic hyponatremia, such as in heart failure, we estimated urine [Na], urine [K], and the amount of urine flow after diuretic administration and included them in the calculation. As it was challenging to predict urine volume at the beginning of the correction, we roughly estimated it. We considered the amount of water and sodium losses via perspiration negligible in typical cases; therefore, we did not take perspiration into account for the predictive correction^38,39^. In cases with a sudden and substantial dilution of urine or rapid increase in serum [Na], desmopressin or hypotonic solution was administered at the discretion of the physicians to prevent overcorrection^15,16,29^. The incidence of ODS, as well as the length of hospital stay and in-hospital mortality rate, were also evaluated.

### Statistical analysis

Categorical and continuous data are presented as total numbers (percentages) and medians with Chi-square test or Fisher’s exact test as appropriate. For continuous variables, we used the Mann–Whitney U test. Logistic regression was used to identify independent potencial factors for appropriate correction of hyponatremia. Known risk factors (younger age, female sex, low BMI, solid tumor, low CCI, low initial serum [Na], low serum [K], low urine [Na], and low urine osmolality) for inappropriate correction and factors that were deemed important (cause of hyponatremia [drug-induced, unidentified cause], daily use of thiazide diuretics, the correction method [Edelman equation], number of serum [Na] measurements during the first 24 and 48 h, isotonic saline, hypertonic saline bolus and continuous infusion, electrolyte repletion, desmopressin, and kidney specialist intervention]) were included with or without *P* < 0.05^28–30^. Separate analyses for undercorrection versus non-undercorrection and overcorrection versus non-overcorrection were also performed. All statistical analyses were performed using SPSS Statistics (version 22; IBM Japan, Tokyo, Japan).

## Supplementary Information

Supplementary Tables.

Supplementary Figure 1.

## Data Availability

All data generated or analyzed during this study are included in this published article.
