# Supplementary figures and images for: Predictive correction of serum sodium concentration with formulas derived from the Edelman equation in patients with severe hyponatremia
Source: Sci Rep. 2023 Jan 31;13:1783. doi: 10.1038/s41598-023-28380-y (PMC9889706; doi:10.1038/s41598-023-28380-y)

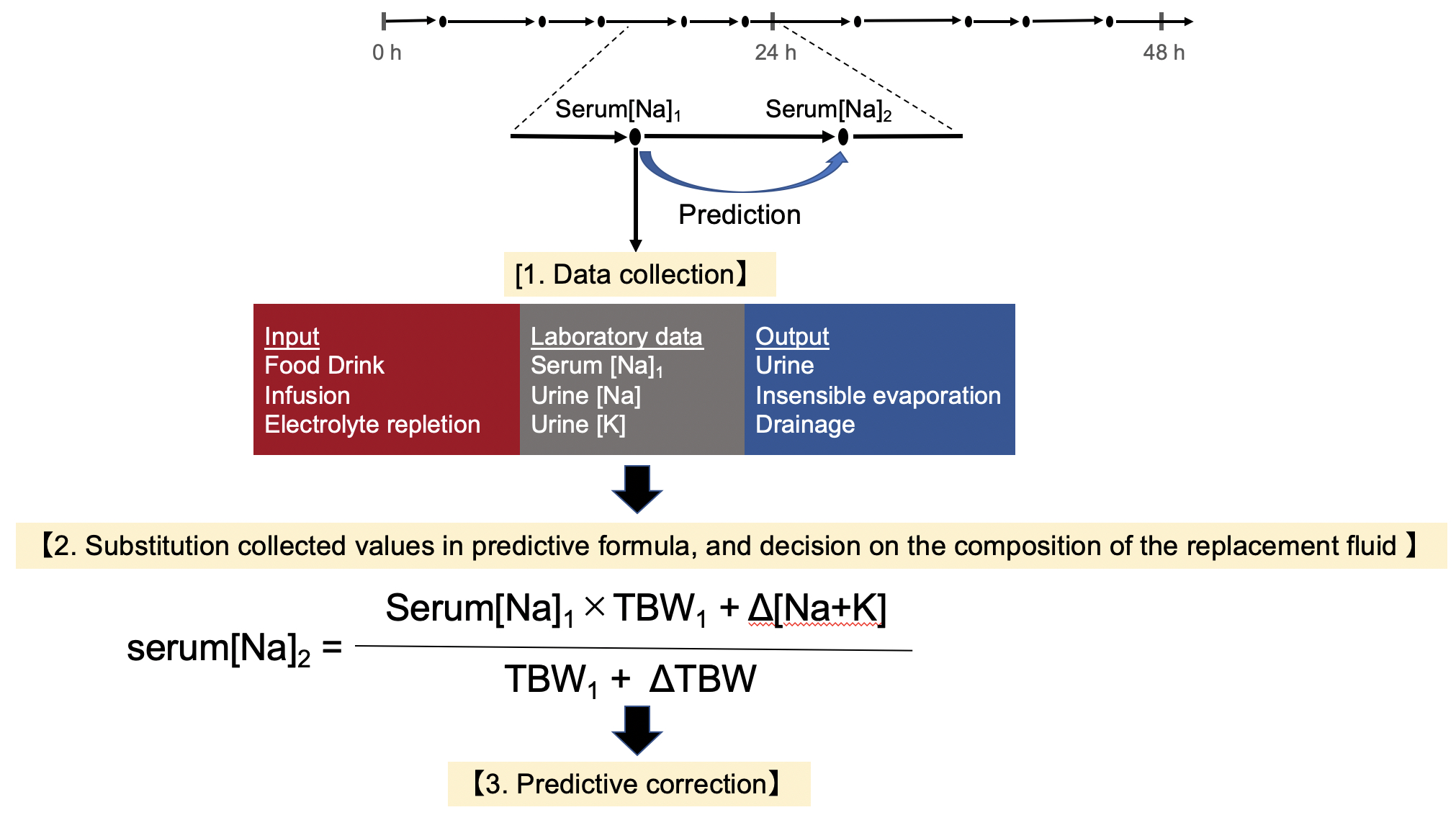

Supplement: Supplementary file 2 — Supplementary Figure 1. [file 41598_2023_28380_MOESM2_ESM.jpg]
